# Editorial: Immunity in Compromised Newborns

**DOI:** 10.3389/fimmu.2021.732332

**Published:** 2021-07-26

**Authors:** Per T. Sangild, Tobias Strunk, Andrew J. Currie, Duc Ninh Nguyen

**Affiliations:** ^1^ Comparative Pediatrics and Nutrition, Department of Veterinary and Animal Sciences, University of Copenhagen, Frederiksberg, Denmark; ^2^ Department of Neonatology, Rigshospitalet, Copenhagen, Denmark; ^3^ Department of Pediatrics, Odense University Hospital, Odense, Denmark; ^4^ Centre for Molecular Medicine & Innovative Therapeutics, Murdoch University, Perth, WA, Australia; ^5^ Wesfarmers Centre for Vaccines and Infectious Diseases, Telethon Kids Institute, Perth, WA, Australia; ^6^ Neonatal Directorate, Child and Adolescent Health Service, Western, Australia; ^7^ Centre for Neonatal Research and Education, The University of Western Australia, Perth, WA, Australia

**Keywords:** immune development, inflammation, antibiotics, preterm, birth, milk, sepsis, necrotizing enterocolitis

The risk of infection-related morbidities and mortality is particularly high in the newborn period. Before birth, the mammalian fetus is protected from adverse effects of exogenous pathogenic microbes and can normally develop its immune system in a near-sterile environment with limited need for immune responses. This condition changes dramatically at birth when rapid adaptations of the innate and adaptive immune systems are required to tolerate and respond to commensal and pathogenic bacteria at epithelial surfaces (e.g. gut, lungs, skin) and fight microbes penetrating to blood and internal organs. Carefully balanced responses of the systemic, organ-related and epithelial immune systems are required to avoid bacterial overgrowth, translocation across immature barriers, and excessive inflammation. The many arms of the mammalian immune system develop differently in different species but comparative studies facilitate insights into mechanisms of perinatal immune development and help identify prophylactic and therapeutic opportunities. Interventions to support neonatal immunity are most critical for those born preterm, growth-restricted, hypoxic, infected or otherwise compromised at birth.

This Research Topic presents a collection of 29 original research articles and reviews on perinatal immunology, aimed to understand the special challenges of compromised newborns. The Research Topic collection is connected with the completion of the international NEOMUNE research consortium, led by University of Copenhagen (www.neomune.ku.dk, 2013-20), having a focus on milk and microbiota influences on gut, immunity and brain development. The Research Topic and this editorial combine knowledge obtained in the NEOMUNE consortium with a series of complementary articles. We encourage studies into the mechanisms of systemic and mucosal immune development, and how dietary, microbial and pharmacological interventions support immune maturation assessed by both classical immune markers and exploratory omics techniques. The latter methods have recently emerged as novel tools to better understand immune development and prepare the way for a new precision medicine approach to the prevention, diagnosis and treatment of neonatal immune disorders (1, 2).

## Transition at Birth and Postnatal Immune Development

Before birth, the uterus and fetal membranes protect the mammalian fetus from exposure to environmental bacteria, viruses and fungi, and the mother is kept in a state of relative immunosuppression to avoid immunological rejection of the fetus. At this time, placental integrity, a near-sterile environment (particularly in the first two trimesters of pregnancy) and maternal immunity protect the developing fetus against infections. After birth, the protective and immunomodulatory properties of colostrum and milk provide continued support, concomitant with a gradual development of both the innate and adaptive arms of the newborn immune system. The innate immune system and the epithelial barriers are the first line of defense against infections and immune cells can react rapidly, non-specifically and pre-programmed to combat infectious stimuli before more adaptive immunity develops. The cellular, structural and functional elements of the immune system may remain distinct from those in older individuals for days, weeks or months after birth, yet this special early life immune status may also confer certain survival benefits for the host. Thus, a relatively ‘dormant’ immune system may support a physiologic and metabolic state that helps to dampen hyper-inflammatory responses following the sudden exposure of the newborn host to a world of microbes (3, 4).

What then if newborns are born too early and/or too small? Annually, an estimated 20 million infants (10-20% of all infants) are born preterm (< 37 weeks gestational age, GA) and/or growth-restricted (< 10% growth percentile). Their health complications account for up to half of all infant deaths (5, 6). Preterm birth is associated with short- and long-term health consequences, including increased infection rate, even until adulthood (7). On the other hand, the immunological adaptation of such compromised newborns in early life is remarkable. Across several papers, this Research Topic demonstrates that mammals have a surprising capacity to adapt their immune systems postnatally, even after serious prenatal insults.

In humans, spontaneous preterm birth is related to one of two overlapping disease etiologies: Fetal infection/inflammation, leading to placental dysfunction, or placental vascular dysfunction, causing hypertensive disorders and fetal growth restriction (with/without preterm birth). Dramatic advances in neonatal medicine, including ventilator management, nutrition and medical therapies now allow good survival rates of extremely preterm infants from 60% GA (of total 40 weeks). Yet, the immature state of many organs at birth predisposes to later complications, many of which have a clear immunological or inflammatory component (e.g. LOS, late-onset sepsis; BPD, bronchopulmonary dysplasia; NEC, necrotizing enterocolitis; WMD, white matter damage; PNALD, liver parenteral nutrition associated liver disease; AKI, acute kidney injury). Understanding the complexity of all these interacting diseases in early life of preterm infants demands a multi-organ research approach and appropriate animal models of immune-related disease in early life.

Successful perinatal immune adaptation is a delicate balance between tolerance and resistance of the immune system to pathogens and/or inflammatory factors, with bacterial colonization along host epithelial surfaces and diet playing important roles. Many endogenous host factors may support the development of balanced immune responses. In this Research Topic, Viemann reviewed how alarmins, e.g., S100A8/9, are crucial factors for perinatal immune regulation. Based on a large cohort of Gambian children, Odumade et al. indicated that developmental changes in adenosine deaminase (ADA) levels may also play a role for this immunological transition. In therapeutic settings, adjunct therapies to antibiotics, such as the phosphodiestarease inhibitor pentoxifylline (PTX), may help to dampen damaging pro-inflammatory responses, without limiting bacterial clearance, as shown by Speer et al. in newborn mice infected with Gram-negative *E. coli*. Similar anti-inflammatory effects in newborns are shown after Gram-positive infection of newborn mice (8), and as shown in this Research Topic by Gravina et al. The size of such pups (1-2 g) makes longitudinal clinical assessment and blood/organ sampling difficult. In this regard, preterm piglets may be more suitable models for recording multi-organ effects of systemic infections and sepsis in compromised infants, as shown previously (8, 9) and in this Research Topic by Bæk et al. Muk et al. showed how serial blood and cerebrospinal fluid (CSF) collections from preterm piglets can be used to reveal new proteome biomarkers of neonatal Gram-positive infection, potentially affecting both systemic and brain compartments, and how responses are modulated by milk diets.

Preterm infants have a T cell population adapted to life in utero, in part explaining their reduced Th1 response and higher susceptibility to infections, but with more systemic Treg cell activity to help protect them from excessive inflammation. In this Research Topic, the reviews by Sampah and Hackam and Sproat et al. add important information to existing reviews on immune ontogeny in infants and include description of the various cellular components (4, 10). The reviews focus on how immature infants show deficient capacity to respond to bacterial and viral infections in blood, organs and/or along surface epithelia.

Neutrophils are a vital component of innate immunity since they are often the first cells to be recruited to fight bacterial, viral, and fungal infections. Neonatal neutropenia is a common phenomenon after preterm birth (11) but neutrophils also have a remarkable capacity to proliferate, adapt and respond to infectious challenges in the days and weeks after preterm birth, as shown in preterm pigs (8, 12, 13). Notable phenotypic and functional disparities exist between neonatal and adult neutrophils with regards to cell membrane receptors and functions. Depressed neutrophil phagocytic function in newborns may function to allow establishment of a healthy microbiome (14). Neutrophils are also essential components for proper B and T cell function, antigen presentation, and tissue repair and regeneration and these neutrophil functions are reduced in preterm neonates with high risk of LOS and NEC (14), similar to many other immune cell types and functions (Sampah and Hackam). Across preterm infants, pigs and newborn mice, there is up-regulation of circulating cell-free DNA and neutrophil-associated proteins at or shortly before LOS and/or NEC (8). However, not all immune cell populations and functions are immature in preterm neonates as discussed in the Research Topic reviews by Sampah and Hackam, and Sproat et al., supporting the observed similar reponses to pathogens by preterm and term blood monocytes (15).

In the intestine, the mucosal innate immunity and repair mechanisms rely on numerous interacting components from the environment (diet, microbiota) and specialized cell types support immune tolerance and sensitivity to inflammatory insults. The review by Leushcow and McElroy provides an example from the perspective of the relatively late-developing intestinal Paneth cells, secreting important antimicrobial peptides. Thus, ablation of murine Paneth cells in growing mice results in greater sensitivity to NEC lesions, and infants with NEC have reduced Paneth cell numbers in their intestinal tissues. Such mucosal effects may have long-term consequences for the immature gut via epigenetic regulation of gene transcription. A study by Pan et al. in this Research Topic shows that the immature intestine has a remarkable capacity to adapt its immune gene expressions soon after preterm birth in pigs. This multi-level control of postnatal innate immunity is critical to avoid excessive gut inflammation to colonizing bacteria after birth, leading to NEC, and its interaction with inflammatory responses at other epithelia or distant organs. On this background, it is not surprising that 10-30% of very preterm infants develop immune-related disorders such as NEC and LOS and that many organ complications in preterm infants are partly related to dysregulated local host immune responses to infection/inflammation. Hyper-inflammatory conditions like NEC and LOS may also induce immunosuppression, predisposing to secondary infection risks, as indicated by infant studies (16) and blood cell transcriptome and immune studies in preterm pigs with NEC (17, 18).

## Adaptive Immunity and Immunization in Compromised Newborns

Systemic immunoglobulin G produced by B cells is the most abundant antibody type in the body, binds to cell surface receptors on many cell types to stimulate immune cell phagocytosis, cytotoxicity and activates complement cascades. Thus, systemic transfer of immunoglobulins from mother to offspring during the fetal and/or neonatal period helps to expose naïve immune cells to antigens in a controlled fashion, regulate their pathogen responses and stimulate maturation of the immune system, facilitate tolerogenic responses and prevent excessive inflammatory responses. Distinct differences exist among mammals in the time and mode of transmission of this passive immunity. In newborn ungulates (e.g. large farm animals) this occurs mainly via uptake of colostrum via the gut while for infants this occurs in utero across the placenta in the last third of pregnancy. The Research Topic review by Westrøm et al. provides an updated review on the knowledge of the immature gut barrier and the transfer of immunoglobulins from mother to young across species, and its role in immune maturation. Such passive immunoglobulin transfer may have effects beyond immune protection in gut and blood, potentially affecting more distant organs such as the immature brain, as reviewed by Pierzynowska et al.

At birth, very preterm infants have <50% of serum immunoglobulin G levels, compared with term infants (19), contributing to their high infection sensitivity. Conversely, preterm newborn pigs or other husbandry animals are completely devoid of blood immunoglobulins making them even more sensitive to systemic infections, even by low-virulent pathogens, if not fed mother’s colostrum, as demonstrated by Bæk et al. In preterm newborns, such infections may lead to inflammatory lesions in various organs, including the immature brain (9). Supplemental systemic immunoglobulin has limited LOS- and NEC-preventive effects in pigs or infants (20, 21). Conversely, protective effects of oral IgG, and especially IgA, are possible (22), particularly for formula-fed preterm infants. Such effects may occur via local gut immune responses to colonizing bacteria, highlighting the importance of fresh maternal milk for compromised newborns (see later section).

Active immunization against specific pathogens in early life is often hampered by impaired initiation, immunogenicity, antibody production and cell-mediated response, requiring highly species-, age- and patient-specific vaccine approaches (23). These concerns of inadequate vaccine responses in newborns may be particularly relevant for preterm infants and Kulkarni-Munje et al. showed that although Indian preterm infants mounted adequate systemic immune responses to the majority of antigens of a pentavalent vaccine, diminished immunological memory remained a concern, relative to term infants. Local tissue environments and mediators from non-immune cells may determine the degree to which individual tissues respond effectively to infections and vaccinations, as shown by Bonney et al. for lung epithelial production of IL-6, supporting immune responses in influenza-challenged young mice. Similarly, in vitro work by Sharma et al. on isolated APC cell lines from mouse lungs, demonstrated how co-stimulants, including a ligand of the viral TLR5 receptor, flagellin, are effective to support cellular responses to viral infections or nasal vaccination in early life. Along such body epithelia, the virome may be critical for early life innate immune ‘training’ of the newborn host. In fetuses and preterm newborns, relatively harmless viral exposures, such as those of cytomegaloviruses (CMVs), may cause serious organ injury, including in the CNS. The tissue- and age-specific interaction between CMV and host cells depends critically on macrophages and monocytes, facilitating the balance between ‘longer-term immune benefits or immunopathology´, as elegantly reviewed by Baasch et al. in this Research Topic. Possibly, diet-dependent effects of the developing gut virome and bacteriome determine later-life mucosal immunity via cell-specific epigenetic changes (24).

Locally in the gut, toll-like receptors are important to recognize pathogen-associated molecular patterns (PAMPs) in early life and could trigger innate immune responses not only in the gut, but also in distant organs, such as the lung and brain, by signaling the recruitment of immune cells to these organs and facilitate secondary immune responses, as reviewed by Sampah and Hackam. Experimentally, this is well demonstrated in mouse pups where NEC-related activation of TLR4 leads not only to systemic immune responses, but also to homing of gut-derived IFN-γ releasing CD4+ T cells to the brain, activation of inflammatory responses in microglial cells (25), or corresponding homing of proinflammatory Th17-secreting cells to lungs, activating local TLR4-dependent pathways (Sampah and Hackam). Consistent with this, brain inflammatory responses were also demonstrated after development of severe NEC lesions in preterm pigs (26, 27). In this Research Topic, Wedgwood et al. showed in young rat pups that the inflammatory effect of postnatal growth restriction on the developing lung may occur partly via changes to the gut microbiota with excessive activation of Gram-negative (TLR4-mediated) pathways. This helps to explain the commonly observed association between gut bacterial dysbiosis and NEC, growth restriction and later lung BPD development in preterm infants.

## Fetal Insults Affecting Newborn Immune Development

Inter-organ immune communication and inflammatory insults may occur even before birth, preterm or term, and affect postnatal immune development. Chorioamnionitis (CA, inflammation of fetal membranes) is a key predisposing factor for preterm birth and adverse postnatal outcomes in CA infants (e.g. EOS, NEC, WMD, BPD) are well known but responses are highly time-, GA-, age-, organ- and pathogen-specific. There are reports of reduced LOS risk following CA in preterm infants (28), and fetal inflammation may under certain circumstances induce immune maturation. The systematic review by Villamor-Martinez et al. shows that the overall moderate CA-associated increase in later infections (LOS, 1-1.5 fold) partly relates to CA infants being born at lower GA while the CA effect on infant infections at birth is clear (EOS, 3-5 fold increases). By transcriptomic analyses on cord blood, Golubinskaya et al. showed high expression of S100A alarmins in cord blood monocytes of CA-exposed preterm infants, potentially helping to diagnose and treat CA effects after birth. Such associations show the need to do well-controlled studies in animals, allowing separation of the effects of preterm birth from its fetal and maternal predisposing factors, and thereby understand mechanisms across species and organs.

Following their previous works on the developing brain and lung, showing highly dose- and time-dependent effects of intra-amniotic inflammation (29), Heymans et al. demonstrated in cohorts of fetal lambs that fetal inflammation induces marked structural changes to the immature gut, including its enteric system and immune-regulatory glial cells. From lamb studies, it remains unclear if such effects predispose to later development of NEC lesions because preterm lambs are difficult to rear postnatally. In preterm pigs, few days of intra-amniotic exposure with LPS (reflecting Gram-negative infection) had limited or no effects on later NEC sensitivity (30, 31), or on gut microbiota or mucosal transcriptome, as observed by Pan et al. in this Research Topic. In this study, any postnatal gut influence of fetal inflammation appeared overshadowed by the immune-modulating effects of enteral feeding and microbial colonization just after birth. On the other hand, Muk et al. showed that kidneys, in contrast to gut, lungs and liver (30), showed more robust and longer-term inflammatory reactions after prenatal Gram-negative infection in preterm pigs, confirming the postnatal systemic immune suppression assessed by blood transcriptome analyses (32). It remains that the immune effects of fetal inflammation are highly species-, time-, age-, pathogen- and organ-specific. While animal studies help to verify mechanisms, it remains challenging to apply this to interventions preventing infections in complex clinical settings. 

Another large proportion of compromised newborns, both preterm and term, are those exposed to placental dysfunction, with or without infection/inflammation, leading to gestational hypertension, fetal hypoxia and intra-uterine growth restriction (IUGR), with differential effects on immune development. Independently of predisposing factors, IUGR infants show increased infection susceptibility, probably due to low blood leukocyte, lymphocyte and macrophage counts just after birth, while specific effects on gut and lung inflammatory disorders (NEC, BPD) are more limited (33, 34). Yet, infants being born both preterm and IUGR are at highest risk for adverse outcomes, especially when combined with perinatal inflammation. In preterm pigs, moderate growth restriction at birth has limited NEC effects (35) but induces immune suppression in the first week, as shown in another study by Bæk et al. but with limited effects later, as shown in another study by Bæk et al. The studies in preterm pigs confirm the remarkable immunological adaptive capacity of immature and compromised newborns (12, 13, 36).

The synergistic effect of inflammation and dysregulated blood and oxygen supply around birth has been used to establish term animal models of perinatal gut, lung and brain damages (37, 38). Gravina et al. demonstrated in mice that the brain-damaging effects of hypoxia were most pronounced in male pups and during the acute phase of systemic infection with gram-positive bacteria. Male-specific higher mortality and immune defects were also demonstrated in the immediate postnatal period of preterm pigs (36).

## Diet and Postnatal Immune Development

The host immune system undergoes the most rapid changes in the perinatal period, together with profound changes in two central immune-modulating environmental factors, diet and microbiota. It is therefore not surprising that even small perturbations in this co-development can have profound consequences, especially for those with an underdeveloped immune system. A critical window of diet-microbiota susceptibility may exist shortly after birth and early life (dietary) interventions may have most pronounced effect on immune development in the first days and weeks of life, especially when birth occurs preterm. Initially, these infants are often nourished parenterally but lack of luminal gut stimulation may in itself lead to impaired mucosal and systemic immune development via deficient gut trophic and immunomodulatory effects (e.g. immunoglobulins, lactoferrin, growth factors) and disturbed bacterial colonization. Further support for early enteral nutrition is related to the risk that excessive supply of parenteral metabolic substrates (glucose, amino acids) may create hyperinflammatory responses in immature immune cells (4).

Breast-feeding associates with fewer infections in infants, but effect sizes vary greatly (39) and are most pronounced within the first year of life (40) and for preterm infants. In this Research Topic, Sproat et al. summarized the factors in milk (immunoglobulins, milk immune cells, oligosaccharides, lactoferrin, growth factor peptides) that may affect innate and adaptive immunity in immature newborns. The first milk, colostrum, is particularly rich in immune-regulating factors and these may act to enhance immune defense in both species-specific and species-unspecific ways. Recently, bovine colostrum was investigated as a supplementary diet for mother´s own milk, formula or donor human milk for preterm infants (41, 42), based on preterm pig studies (9, 35, 43–48). In this Research Topic, Li et al. and Bæk et al. showed that bovine colostrum feeding before or after formula feeding had greatest effects on both mucosal and systemic immunity in preterm pigs when provided immediately after birth (e.g. blood neutrophils, Treg cells, gut IL8 response). Further, Pan et al. demonstrated epigenetic programming and NEC-protective effects of early colostrum supplementation on gut mucosal immunity and gene expression in newborn preterm pigs on parenteral nutrition (44, 49). In either case, the effects may be mediated by local species-unspecific immunoglobulin binding of pathogens and modulation of local mucosal immune responses (50, 51). The potential to use bovine colostrum as supplemental immunological protection in compromised preterm infants and other sensitive pediatric groups, is currently being investigated (41, 42, 50, 52).

## Microbiota, Antibiotics and Newborn Immune Development

A wealth of information has recently emerged on the role of the developing gut, lung and skin microbiota on immune development in compromised newborns. While many studies in infants report only associations, not cause-effect relationships, direct effects of pre-, pro and antibiotics (AB) on the developing immune system via changes to the gut microbiota are demonstrated across many studies. However, due to wide individual variation in gut microbiota composition, type of pre- and probiotic products, and their interactions with milk diet and the non-bacterial gut microbiota (viruses, fungi), many questions remain unresolved. Across trials and products tested, dietary pre- and probiotics reduce NEC, and to a lesser degree, LOS in preterm infants (53, 54). Increased local and systemic immune competence after pre- and probiotic exposures may occur via competitive exclusion of pathogens, production of antimicrobial products and activation of mucosal immune cells with subsequent systemic effects. Finally, benefits may also occur by enhancing gut barrier properties, especially considering that a large proportion of LOS cases result from translocation of dominating gut bacterial species across an immature gut (55, 56). Similar barrier mechanisms may explain why rectal (not oral) transplantation of a mature fecal microbiota protects against NEC and mucosal bacterial adherence in preterm pigs, while stimulating systemic immune cell populations (57).

Due to the risk of systemic infections at or shortly after birth, empiric use of broad-spectrum AB is common for newborn very preterm infants [50-100% of infants (58),]. At population levels, long-term use of AB is clearly associated with adverse immune outcomes, gut microbiota turbulence, and increased antimicrobial resistance (4). The study by Oosterloo et al. in this Research Topic shows that immune-related markers in plasma of term infants may be affected up to 1 year after neonatal exposure to AB. In piglets, Hu et al. showed that low-dose, longer-term AB treatment reduced Th1-related blood immune responses while intestinal innate immunity-related genes were enriched, together with reduced IFN‐γ and IL‐6 expression. Together these studies indicate that AB-treated hospitalized infants could be more sensitive to later gut and systemic infections, especially if treated for longer periods (59). Importantly, ‘confounding by disease’ often complicates interpretation from such studies, and effects may vary according to time, dose, duration and route of AB administration. In very preterm infants, short-term (< 3 days) neonatal systemic AB treatment was associated with less (not more) NEC, but effects on systemic immunity or gut microbiota were unknown (60). In this Research Topic, Jiang et al. showed by plasma proteomics that NEC progression in preterm pigs affected many systemic immune markers, while short-term systemic AB treatment (< 5 days) had limited effects. On the other hand, neonatal AB treatment by the oral route seemed to delay gut colonization, improve gut immune gene expressions, plasma proteins and metabolites, indicating immune maturation and reduced systemic inflammation (56, 61–64). In immature, compromised newborns, an initial delay in gut bacterial colonization may allow better control of immune development but these initial benefits may later reduce both mucosal and systemic immune defense in both preterm pigs and infants (4, 59, 65). More research is required to demonstrate both benefits and possible harm of early life microbiota interventions for various subgroups of compromised newborns.

## Conclusions

Birth is a dramatic, yet miraculous event. Rapid adaptations are required throughout the body to survive the transition from a stable life in utero to the microbe-dense outside world. These adaptations include tolerance to billions of colonizing and invading bacteria, viruses and fungi along the outer surfaces (gut, lung, skin), together with completely new modes of nutrition, respiration, metabolism and excretion. Via 29 reviews and original research articles this Research Topic has shown how perinatal immunity develops in compromised newborns. Dysregulated immune development in newborns born preterm, growth-restricted, infected or subjected to placental dysfunction or birth asphyxia - is not a surprise. Rather, it is surprising how well these infants survive, adapt and thrive, despite their increased susceptibility to infections and inflammation in early life. Due to the age-, cell–, organ- and species-specificity of the developing immune systems, only a few interventions enhancing bacterial protection and immunity have become universally accepted for sensitive infants (e.g. breast-feeding, hygiene, certain antibiotics, vaccines). Novel dietary, microbiota and pharmacological interventions still require better documentation and evidence of mechanisms. Cross-species studies and omics-based analyses may help to understand mechanisms and ensure healthy development without side effects. Newborns that are compromised by fetal growth restriction, inflammation, infection, preterm birth and/or delivery complications often experience immunological deficits in the immediate postnatal period. Yet, their adaptive capacity is remarkable, making immune modulation of compromised newborns a difficult balance between potential benefits and possible harm.

## Author Contributions

PS, TS, AC and DN served as Guest Editors in the Research Topic. PS wrote the outline of this manuscript. TS, DN and AC reviewed and edited the manuscript. All authors contributed to the article and approved the submitted version.

## Funding

The work was funded in part by grants from Innovation Fund Denmark (NEOMUNE and NEOCOL grants).

## Conflict of Interest

The authors declare that the research was conducted in the absence of any commercial or financial relationships that could be construed as a potential conflict of interest.

## Publisher’s Note

All claims expressed in this article are solely those of the authors and do not necessarily represent those of their affiliated organizations, or those of the publisher, the editors and the reviewers. Any product that may be evaluated in this article, or claim that may be made by its manufacturer, is not guaranteed or endorsed by the publisher.
